# Aging of the Human Lip: Current Knowledge and Clinical Implications

**DOI:** 10.1111/jocd.70310

**Published:** 2025-07-30

**Authors:** Fanghui Sun, Yue Liu, Tao Zhang

**Affiliations:** ^1^ R&D Center, Better Way (Shanghai) Cosmetics Co., Ltd Shanghai People's Republic of China

**Keywords:** anti‐aging approach, lip aging, molecular mechanism, skin barrier, skin physiology/structure

## Abstract

**Background:**

Comprehensive exploration of lip aging remains limited due to anatomical complexity of the structure, with current investigations predominantly focusing on aspects such as dryness management, cheilitis prevention and treatment, and surgical lifts.

**Aims:**

This article aims to review multiple aspects of lip aging and to explore anti‐aging approaches for the lips, thereby providing actionable insights for the development of cosmetic lip care products.

**Methods:**

We conducted a literature search in the PubMed database using keywords related to lip aging, and selected the most relevant articles for in‐depth reading, summarization, and integration to form the content of this review.

**Results:**

Due to the significant differences between lip and facial skin in terms of barrier function, melanin content, transepidermal absorption capacity, physiological activity frequency, and fat distribution, the aging characteristics of lips also exhibit uniqueness. At the same time, there are gender differences in lip aging manifestations, with women typically showing more noticeable perioral wrinkles compared to men.

**Conclusions:**

The clinical manifestations and pathogenic mechanisms of lip aging differ significantly from those of facial aging, requiring distinct priorities in anti‐aging product development. For the vermilion, the focus should be on barrier restoration, mild anti‐aging approaches, and photoaging prevention. In contrast, perioral anti‐aging may be addressed by inhibiting ECM degradation and enhancing tissue volume. Addressing gender differences can guide the development of anti‐aging lip products tailored to women, while managing associated pigmentary changes offers a unique avenue for this purpose.

## 1 Introduction

The lips, situated in the lower central part of the face, serve essential functions such as eating, speaking, and expressing emotions. Full, plump, and rosy lip states are crucial for the overall aesthetics and youthfulness of the face, making it a significant functional and aesthetic feature. Similar to other body parts, the lips undergo aging; however, lip aging signs are more immediately noticeable, leading to a growing consumer interest in lip anti‐aging solutions. Owing to its particular physiological characteristics and location, research on lip aging is more intricate than that on other skin regions and remains in its nascent stage. The majority of studies merely focus on specific aspects, such as dryness and moisturization of the lip, cheilitis and its prevention, and lip surgical lifting, with a dearth of systematic research and discussion regarding lip aging and anti‐aging strategies that are of utmost concern to consumers.

Utilizing PubMed as the principal database, we conducted a literature retrieval using the keyword “facial aging” and found a substantial body of work with 17 982 articles. However, when we searched the literature using “lip aging,” the number of studies was significantly lower, with only 1052 articles highlighting that lip aging has not been as extensively researched as facial aging. These studies included many related to aesthetic medicine. Despite the effectiveness of aesthetic medical interventions for lip rejuvenation, their invasiveness, high costs, and potential safety risks are inconsistent with mainstream consumer preferences, which lean toward lip care products for daily use. Therefore, we excluded the literature related to aesthetic medicine and focused instead on the clinical aspects, potential mechanisms, and gender differences in lip aging. Consequently, we conducted targeted searches using “perioral aging” and “vermilion aging” as key terms, systematically reviewing and integrating over 120 relevant studies, including original research and review articles.

Based on the retrieved literature, this study aimed to provide a comprehensive overview of the definition, clinical manifestations, onset timing, gender differences, and mechanisms of lip aging. In our review, we discovered that lip and facial aging differ, necessitating different focuses and considerations in the development of anti‐aging products. Here, we summarize the scientific basis for the differences between lip and facial aging and discuss the focus and considerations in developing anti‐aging lip products based on these differences. Furthermore, we reflected on the current gaps in lip‐aging research and offered perspectives on future research directions.

## 2 Basic Structure of the Lip

**FIGURE 1:**
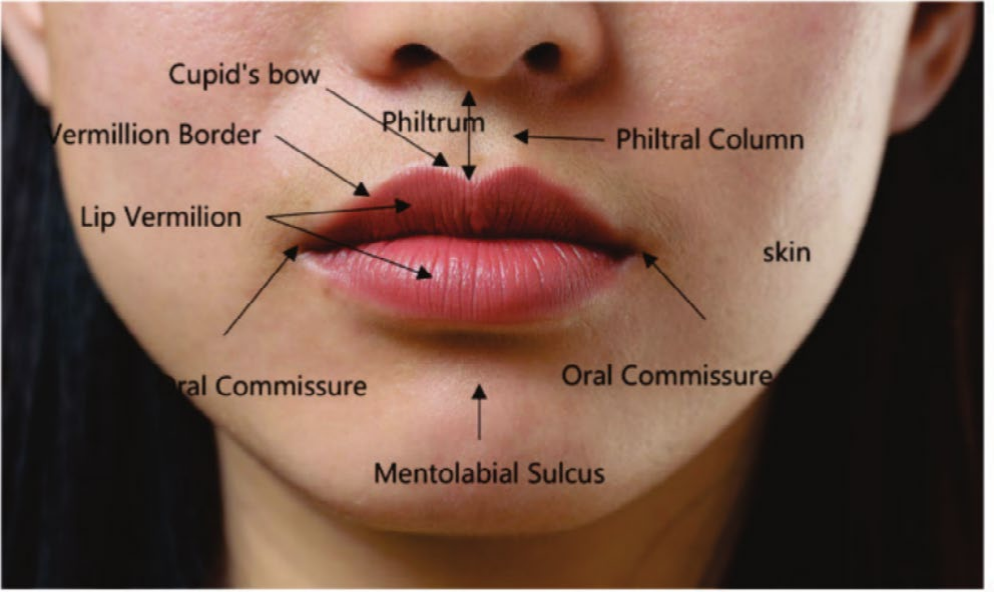
Basic structure of the lip [2].

The upper lip extends inferiorly from the base of the nose to the mucosa, and laterally to the nasolabial folds. The two‐layered structure in the middle is called the lip vermilion, consisting of a non‐keratinized lamellar squamous epithelium, and varies in color from reddish pink to brown, depending on ethnicity. The lower lip extends from the lower margin of the lip vermilion down to the mandible and laterally to the oral commissure. The vermilion border of the upper lip presents the Cupid's bow peaks and valleys due to the traction of the levator labii superioris muscle, thus presenting an arcuate shape known as the “cupid's bow.” The cylindrical depression above the Cupid's bow is a philtrum, and the vertical protrusions on both sides are philtral columns [1]. In addition to the lip vermilion skin, which comprises a mucoid‐like lamellar squamous epithelium, the skin of the lip is covered by normal skin called white lips (Figure 1).

The lip vermilion represents the histological transformation of the facial skin and oral mucosa, and is a hairless, highly vascularized, non‐keratinized lamellar squamous epithelium. The skin of the lip vermilion, with 3–5 cellular layers, is very thin compared with the typical facial skin, which has approximately 16 layers. In addition, the lip vermilion lacks typical skin appendages, such as hair follicles, sweat glands, and sebaceous glands [3, 4]. The characteristic redness of the lip vermilion is caused by a combination of lower melanin levels, thinner epithelial cells, and an abundance of capillaries under the skin.

**FIGURE 2:**
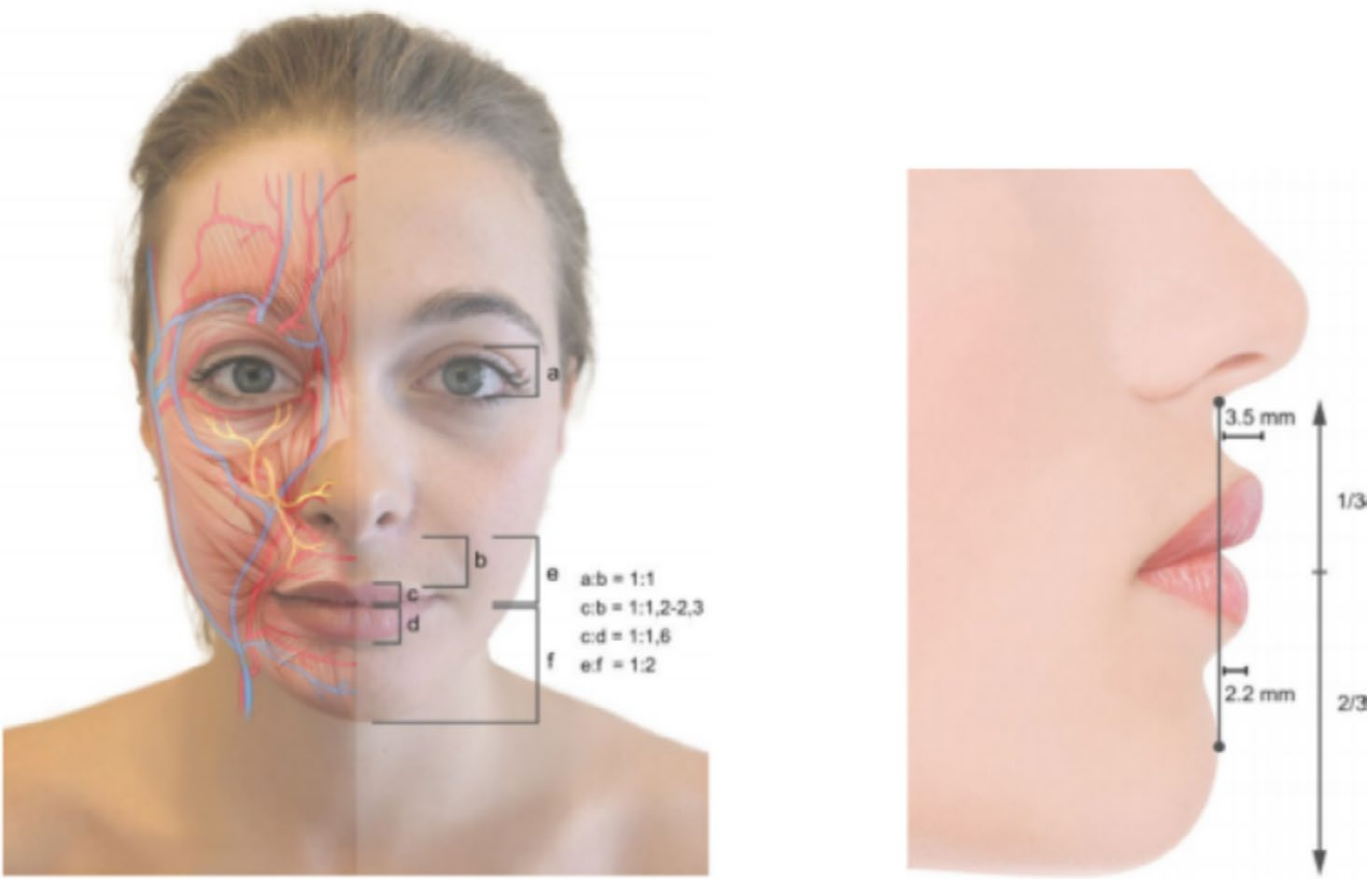
Morphology of young lips [5].

Young lips are usually symmetrical on both sides, with a rosy color, full and elastic, clear vermilion border, smooth lines, no wrinkles, and slightly upturned corners. A short upper white lip is a criterion of beauty and youth, with 7–9 mm the average height of young lip vermilion, while the ratio of the upper red lip to the lower red lip should be 1:1–1.6, the ratio of the total upper lip to the total lower lip should be 1:2, the upper lip should be slightly more projected than the lower lip, and the upper lip should be 3.5 mm in front of a virtually vertical line joining the chin to the sub‐nasal fold, and the lower lip should be 2.2 mm in front of this line [5] (Figure 2).

## 3 Definition of Lip Aging

Anatomically, the lip refers to the region extending from the base of the nose to the lower jaw and laterally to the oral commissures, including the lip vermilion and white lips, and constitutes approximately one‐fourth of the facial area. However, in common parlance, the term “lip” is often used in a more restricted sense to denote the vermilion zone exclusively, with the surrounding area being designated as the “perioral region.” Within the scope of this discussion, “lip aging” predominantly refers to the aging of both the lip vermilion and perioral region; when necessary, we will describe the vermilion border and perioral region separately.

## 4 Clinical Manifestations of Lip Aging

### 4.1 Morphological Change

**FIGURE 3:**
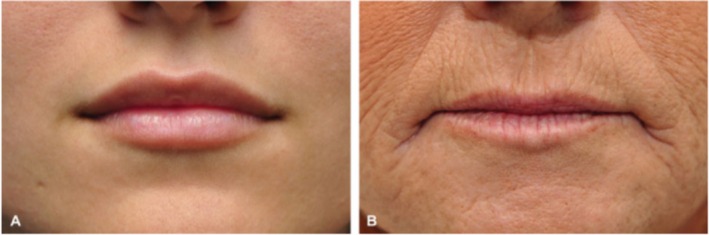
Young and old lips [1].

Bone absorption, soft‐tissue loss, and muscle atrophy of the lips can lead to visible morphological changes. A thinner vermilion is the most obvious change associated with lip aging; the philtrum is lengthened [2], and the lip prominence is decreased; the Cupid's bow widens, and the bow shadow disappears, losing its definition; the areas where the philtrum and philtral columns were located became flat, and the protrusions and depressions were no longer evident; overactivity of the depressor anguli oris muscle leads to downward curvature of the oral commissure, forming prominent marionette lines; the nasolabial groove further deepened due to a decrease in the perioral volume and zygomatic fat pad; and atrophy of the supporting ligament of the labio‐mandibular groove and a downward shift of the fat pad lead to a labio‐mandibular groove deformity (Figure 3).

### 4.2 Surface Change

Owing to sun exposure and other external factors, the degradation of skin elastin and collagen results in deteriorated elasticity and sagging of the lip skin. In addition, daily activities such as showing facial expressions, speaking, chewing, and smoking can all cause the orbicularis oris muscle to pucker. Each time this muscle is used, a groove forms beneath the skin surface. As the skin ages, it loses its elasticity and can no longer return to its original position, causing collagen fibers to break. These grooves become permanent features: the lip wrinkles visible to the naked eye [6].

### 4.3 Faded/Dull Lip Color

The unique position of the lips and complex causes of the vermilion make it highly susceptible to abnormal pigmentary changes. With age, the vermilion color becomes lighter [7], and the border is less defined. In addition, due to the decreasing content of melanin and lower number of melanocytes in the vermilion [8], the skin becomes more susceptible to the effects of ultraviolet (UV) radiation compared to facial skin. This susceptibility induces dysregulated pigment deposition (including melanin accumulation and uneven pigmentation), combined with reduced blood circulation, jointly leading to the dullness in the color of aging lips.

## 5 Lip Aging Onset

Lévêque et al. studied the number of small radiating wrinkles on the upper left side of the lips in 100 female participants aged 20–80 years. The average visibility (length, depth, and width) of the wrinkles was scored on a scale of 1 (not very visible) to 3 (very visible). The “global score” was defined as the product of the number of wrinkles based on their visibility. The results indicated that both the number and visibility of wrinkles showed a strong linear correlation with age, and the global score exhibited a parabolic evolution with age. According to the regression equation of the “number” and the “visibility” of the wrinkles versus age, the first lip wrinkle would appear at 32–33 years of age and become apparent at age 45, which usually corresponds to the start of menopause in females. That is, lip wrinkles begin to appear during the fourth decade, becoming visible during the fifth decade [9].

## 6 Mechanism of Lip Aging

### 6.1 Tissue‐Level Mechanisms

#### 6.1.1 Orbicularis Oris Atrophy

Penna et al. showed that young orbicularis oris have muscle bundles surrounded by a thin layer of connective tissue. As aging progresses, the orbicularis oris shows signs of atrophy, the muscle bundle becomes smaller, and the surrounding epimyosal layer increases. Young orbicularis oris has the shape of “J,” whereas the aged one resembles an “I” [10]. Orbicularis oris atrophy often leads to perioral wrinkles with the oral fissure at the center and radial distribution, which are more likely to occur in whistlers and smokers. Further studies by Gomi et al. showed that a part of the orbicularis oris extending to the vermilion contains fast and slow muscle fibers mainly composed of myosin heavy chain (MYH) −2, −4, and −7. Compared with young people, the expression of MYH‐2 and MYH‐7 in the vermilion of old people was reduced, whereas there was no significant difference in MYH4 expression. Thus, decreased MYH‐2 and MYH‐7 may be responsible for the atrophy of the orbicularis oris, leading to cheilogramma and perioral wrinkles [11].

#### 6.1.2 Bone Resorption

With age, the skeletal structure of the mandible atrophies and alters the underlying support framework covered by soft tissue, which jointly contributes to notable alterations in facial shape [12]. Bone resorption of the maxilla leads to posterior migration of the upper lip position, decreased protrusion of the vermilion, thinning of the vermilion, and changes in the vermilion border curve, presenting an aged appearance [13].

#### 6.1.3 Fat Change

Facial superficial fat is anatomically divided into the nasolabial, malar, orbital, forehead, and jaw fat compartments. Superficial fat drops owing to gravity and repositioning, leading to atrophy because of imbalanced volume changes between the superficial and deep fat compartments. Due to the sagging of the skin and zygomatic fat pad, atrophy of the deep fat inside the cheek, and changes in fat composition, some aging features appear in the perioral area, such as the nasolabial fold becoming deeper and longer and the mandibular region being depressed [14]. However, Penna et al. found an increase in adipose subcutaneous tissue in old lips, which has not been described in the literature. This may be related to the generally higher percentage of body fat in elderly individuals or to the fact that an increase in subcutaneous fat compensates for the decrease in dermal thickness [10]. Therefore, the effects of changes in perioral fat on lip aging need to be further explored.

### 6.2 Molecular‐Level Mechanisms

#### 6.2.1 Degradation of Collagen and Elastin

Collagen and elastic fibers provide structural support to the skin, maintaining its fullness and elasticity. Consequently, the breakage of these fibers reduces the elasticity, prominence, and skin thickness of the lip, resulting in a thinner vermilion, perioral wrinkles, and other aging manifestations [10, 11].

#### 6.2.2 Hyaluronic Acid (HA) Degradation

Skin aging is related to the loss of skin moisture, and the key molecule for skin moisture is HA. HA is a glycosaminoglycan with a unique ability to bind and retain water molecules and is a major component of the extracellular matrix (ECM), which makes the skin plump and elastic [15]. Gomi et al. reported an age‐dependent decrease in HA levels in the upper lip vermilion. Further studies have shown that this decline is caused by the combination of a decrease in HA synthetase 1 (HAS1) and an increase in the enzyme involved in HA degradation (CEMIP, a cell migration‐inducing protein) [11].

#### 6.2.3 Role of Oxidative Stress in Lip Aging

Less melanin in the lips compared with that in the face makes the lips more susceptible to photoaging. Photoaging is driven by the excessive production of exogenous reactive oxygen species (ROS) and their downstream effects. One identified pathway is that increased ROS‐stimulated UV light induces the activation of protein‐1 (AP‐1) and nuclear factor kappa B. These transcription factors induce the production of matrix metalloproteinases (MMPs) and inflammatory cytokines, which degrade the local ECM. Dermal fibroblasts AP‐1 increases the levels of CYR61 (cysteine‐rich angiogenic factor 61, a marker of oxidative stress). CYR61 not only inhibits the production of collagen I, but also promotes its degradation by upregulating the production of MMP1 [16].

#### 6.2.4 Vascular‐Related Changes

Tamura et al. studied the relationship between blood flow in the vermilion and aging using noninvasive spectral reflectance. The results showed that the hemoglobin levels in the vermilion decreased with age [17]. Using histological and anatomical methods, Gomi et al. showed that both the area and the number of blood vessels relative to the vermilion surface length in the dermis of the upper lip vermilion decreased with age [18]. Research by Jin et al. indicates that the number and size of blood vessels in the dermis of Korean people gradually decrease with decades of UV exposure. This may be due to ECM degradation caused by long‐term UV exposure, which destroys the vascular support network, and the damage to blood vessels from persistent chronic inflammation, ultimately leading to vascular regression, sparsity, and disappearance [19]. These studies explain why lip color fades with increasing age.

## 7 Differences Related to Lip Aging

### 7.1 Gender Differences in Lip Aging

Many studies have shown that women exhibit more and deeper wrinkles in the perioral region compared to men [6, 16, 20, 21, 22]. In a clinical study, 33.3% of women were classified as “severe” on a wrinkle scale compared with just 6.6% of men [6]. Therefore, women should pay more attention to lip anti‐aging care. Gender differences in perioral wrinkles may be related to several factors.

#### 7.1.1 Tissue Level

1. The orbicularis oris, which surrounds the lips, is anchored 1.5 times closer to the dermis in women than in men. Fibrous connections between the muscle and the dermis can cause inward traction, thereby creating deeper wrinkles [6].
2. The dermis of the perioral skin in women features a lower number of sebaceous and sweat glands, and the hair follicles are smaller. Therefore, the gross volume of appendages in women is significantly lower compared to men. These appendages stretch fibroblasts and lead to collagen synthesis, with larger hair follicles providing stronger support to the dermis and acting as fillers. This could influence the natural filling of the dermis, making the skin appear fuller and less wrinkled. Therefore, women may be more prone to wrinkling [21, 22].
3. The ratio of the area containing blood vessels and that of connective tissue in the dermis of the perioral skin in men is almost three times higher than that in women. Better vascularization may have a decelerating effect on the development of perioral wrinkles [20].
4. Studies have confirmed that the incidence of hypokeratosis is significantly higher in women than in men because of the thicker stratum corneum of the perioral skin [20]. Because parakeratosis is the main reason for poor barriers, women are more prone to dry aging around the mouth than men.

#### 7.1.2 Molecular Level

1. Brown et al. showed that the average expression of CYR 61 in the perioral skin was 3.5 times higher in women than in men, indicating that there were more senescent dermal fibroblasts and that their ability to maintain dermal matrix structure was relatively weaker [16].
2. Another hypothesis suggests that stem cells for hair follicle in the perioral region are highly active in men, with increased cell migration and turnover. This could promote cell proliferation and the apoptosis of aging cells, thereby delaying aging and reducing wrinkle formation. However, Brown et al. found no significant differences between men and women in the expression of insulin‐like growth factor (IGF‐1), which sustains the growth phase of hair follicles. Moreover, IGF‐1 production declines faster with age in men compared to women. These findings indicate that the hair follicle proliferation hypothesis may not be the primary cause of gender differences in perioral wrinkles, and further research and data are needed [16, 21].

#### 7.1.3 Hormonal Level

Women aged > 45 years have more severe perioral wrinkles, and this has been associated with menopause. During menopause, the decline in ovarian function leads to a significant decrease in hormone levels, resulting in a 40% drop in sebum levels in the skin, which in turn results in more severe perioral wrinkles [20].

### 7.2 Differences Between Lip and Facial Aging

#### 7.2.1 Weaker Barrier Function

Given their unique physiological characteristics and location, high‐frequency conductance (a parameter of surface hydration) on the lips is significantly lower than that on the cheeks. The transepidermal water loss (TEWL) in the cheeks was much higher than that in the rest of the body, whereas the TEWL in the lips was almost three times higher than that in the cheeks, and the TEWL dropped more sharply on the lips than on the cheeks with age [23]. This indicates that the skin barrier function on the lips is worse than that on the face, and water retention is weaker; therefore, the lips often become dry and susceptible to irritation from external factors. Dryness can reduce lip elasticity and make the lip wrinkle more pronounced, whereas external factors can stimulate lip aging via various pathways [24, 25]. There are two possible reasons for this discrepancy.

1. Keratin differences

The keratin density in the lip vermilion decreased, with a gradual decline in cytokeratin (CK) 4, CK 13, and CK 19 on the transition side of the mucosa, as well as in CK 1 and CK 10 on the transition side of the skin. CK 5 and CK 14 are consistently expressed at basal levels and exhibit variable supra‐basal expression. At the vermilion and the oral mucosa junction, the expression of profilaggrin, loricrin, and filaggrin disappears, leading to a change in the phenotype of the laminar squamous epithelium covering the vermilion [2]. The outer layer of the keratinized epithelium consists of dead cells rich in keratin with a strong barrier function, whereas the outer layer of the non‐keratinized epithelium consists of living cells lacking keratin with a weaker barrier function. The epithelial cells in the vermilion are larger than those in the cheek skin, and the keratin content is reduced, resulting in a high incidence of keratosis; therefore, the barrier function in the lip vermilion is weak [2, 23].

2 Lipid differences

**FIGURE 4:**
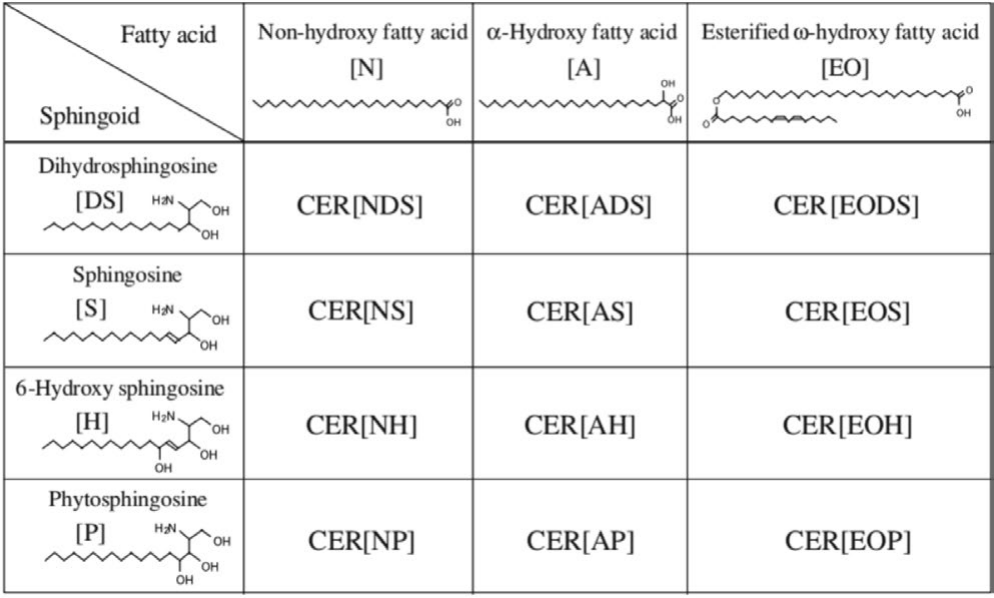
Structure and nomenclature of human SC CERs. CER[NDS] contains non‐OH fatty acids [N] and dihydrosphingosines [DS], CER[NS] contains [N] and sphingosines [S], CER[NH] contains [N] and 6‐hydroxy sphingosines [H], CER[NP] contains [N] and phytosphingosines [P], CER[ADS] contains z‐OH fatty acids [A] and [DS], CER[AS] contains [A] and [S], CER[AH] contains [A] and [H]. CER[AP] contains [A] and [P], CER[EODS] contains ester‐linked fatty acids, ω‐OH fatty acids [EO] and [DS]. CER[EOS] contains [EO] and [S]. CER[EOH] contains [EO] and [H], and CER[EOP] contains [EO] and [P]. These classes are further subdivided into species according to chain length. CER[EODS] could not be detected [28].

Free fatty acids (FFAs), ceramides (CERs), and cholesterol are the main lipids in the skin. They exhibit active metabolism and have an important impact on skin function [26], especially in the maintenance of the skin barrier. Compared with the facial skin, the lip vermilion has extremely few sebaceous glands, and hair follicles and sweat glands are absent. Therefore, it cannot secrete sufficient sweat and sebum to maintain the smoothness and moisture content of the vermilion. Studies have shown that the distribution of FFAs (oleic, palmitoleic, arachidonic, linoleic, and docosahexaenoic [DHA] acids) in the vermilion differs from that in other parts of the skin, with higher accumulation of DHA [27]. CERs, constituting 30%–40% of stratum corneum lipids by mass, play a pivotal role in maintaining epidermal barrier functionality. CERs have been classified into 12 groups according to their fatty acid and sphingoid structures (Figure 4), with further subdivision of each class into species according to the chain length [28]. Research by Junko Ishikawa et al. has shown that the CER spectrum varies according to body regions. Compared with other skin regions of the body, lips have lower levels of total CER, a higher percentage of CER[NS] and CER[AS], a lower percentage of CER[NP] and CER[NH], a lower percentage of CER[NS] with long chains having large total carbon numbers, and a higher percentage of CER[NS] with short chains having small total carbon numbers [29]. A significantly higher distribution of cholesterol sulfate was observed in the superficial skin of the vermilion [26]. The difference in lipid composition led to a higher TWEL, weaker water retention, and poorer barrier function in the lips.

#### 7.2.2 Less Melanin Content

Vachiramon et al. showed that in the vermilion, the ratio of keratinocytes to melanocytes was 10–15:1, while in the facial skin, the ratio of the two was 4:1 [8]. The amount of melanin produced in the vermilion was much lower than that in the facial skin. Melanin is a photoprotective pigment that protects the body from UV radiation and indirectly clears ROS formed during UV‐induced skin oxidative stress [30]. Because the melanin content of the lip is lower than that of other parts of the skin, it is more susceptible to the influence of UV light to produce photoaging, resulting in aging appearances, such as wrinkles, relaxation, and decreased elasticity.

#### 7.2.3 Higher Transdermal Absorption

The lips mainly absorb external substances through transcellular and intercellular routes owing to the absence of skin appendages [4]. Because the lip epithelium contains only three to five cell layers, external substances are more likely to penetrate the stratum corneum and reach deeper skin tissues. The vermilion epithelium is a non‐keratinized squamous epithelium with a weaker barrier function and higher permeability. In addition, the capillaries under the lip skin are abundant and highly perfused, and external substances smeared on the lips can readily access blood circulation through subcutaneous capillaries. The skin barrier of the lips is further weakened when dry and inflamed, resulting in higher transdermal absorption of external substances than in a healthy state. Higher transdermal absorption makes it easier for irritating substances in food, pollutants in the environment, and some skincare ingredients used on the lips to enter deep skin tissue and blood circulation, making it more prone to cheilitis, which may cause inflammatory aging and post‐inflammatory pigmentation.

#### 7.2.4 More Frequent Physiological Activities

Lips perform important physiological functions such as expression, chewing, speaking, smoking, and whistling; therefore, the skin and muscles of the lips move and stretch at a higher frequency than the facial skin, creating dents on the surface of the skin. Over time, the dents become irrecoverable, forming visible wrinkles. During the eating process, oil, salt, pepper, food additives, and other ingredients in food continuously irritate the lip skin. In addition, nicotine and chemicals in lipstick stimulate the lip skin, resulting in aging features such as smoking lines, wrinkles, and lip dullness [31].

#### 7.2.5 Less Subcutaneous Fat

There is less subcutaneous fat in the lips, and the skin and mucosa are almost directly connected to the muscles, thus making perioral wrinkles more obvious [5].

Table 1 summarizes the physiological differences between the lips and facial skin, which lead to significant differences in clinical manifestations of aging between the lips and the face.

**TABLE 1:** Summary of differences between the lips and the face.

			Lip vermilion	Perioral area	Face
Number of cell layers			3–5	N/A	16
Stratum corneum thickness			Thinness	Thickness	Thickness
Keratin density			Low	High	High
Incidence of parakeratosis			High	Low	Low
NMF (tape stripping)			Low	High	High
Skin appendages			None	Have	Have
Sebum content			Low	High	High
Sebum composition	Free fatty acids (DHA)		High	Low	N/A
	Cholesterol sulfate		High	Low	N/A
	Ceramide [28]	Total ceramide level	Low	N/A	High
		CER[AS], CER[NS]	High	N/A	Low
		CER[NH], CER[NP]	Low	N/A	High
TEWL			High	Low	Low
Skin barrier function			Weak	Moderate	Strong
Moisture content			Low	High	High
Transepidermal absorption rates			High	Low	Low
Keratinocyte to melanocyte ratio			10–15:1	N/A	4:1
Melanin content			Low	High	High
Subcutaneous fat content			Low	Low	High
Physiological movement frequency			High	High	Low
Special exposure			Expression, chewing, speaking, smoking, drinking, whistling, lip licking, lip care products, makeup, oil, salt, and chili in food	Expression, chewing, speaking, smoking, drinking, whistling, skincare products, makeup, oil, salt, chili in food, and medical aesthetics	Skincare products, makeup, and medical aesthetics

Abbreviations: CER, ceramide; N/A, not applicable; NMF, natural moisturizing factors; TEWL, transepidermal water loss.

## 8 Lip Anti‐Aging Approach

Lip skin is a type of facial skin; hence, common methods for combating facial skin aging also apply to the aging process of the lips. However, as mentioned in the previous paragraph, the lip skin exhibits different characteristics compared with the facial skin; consequently, more attention should be given to gentle anti‐aging strategies when developing anti‐aging lip strategies. Skin barrier repair is a targeted anti‐aging strategy for lip aging.

### 8.1 Gentle Anti‐Aging

The physiological characteristics of the lip are similar to those of sensitive skin, rendering the lip inherently sensitive and more susceptible to external stimuli. Therefore, it is imperative to implement gentle anti‐aging strategies. When developing anti‐aging products for the lip, it is important to select mild, non‐irritating, and naturally derived cosmetic ingredients to avoid the use of harsh anti‐aging ingredients such as retinol.

#### 8.1.1 Retarded Photoaging

The diminished melanin content in the lip vermilion necessitates a heightened focus on photoaging protection in lip vermilion anti‐aging strategies compared with facial anti‐aging regimens. A sun‐protective agent can be added to lip‐care products to reduce UV and blue light exposure. In developing lip‐sun protection products, a balance between sun protection factor (SPF) and mildness should be considered rather than solely pursuing a high SPF. Additionally, the antioxidant defense system of the lips can be strengthened to reduce ROS and avoid damage to DNA, proteins, lipids, and other macromolecules caused by oxidative stress. Compounds like vitamin C (ascorbic acid) and vitamin E (tocopherol), as well as their derivatives, are known for their potent antioxidant properties and serve as effective antioxidants. Factors related to the signaling pathway can be targeted to block the transmission of the photoaging signaling pathway and avoid subsequent harmful effects.

#### 8.1.2 Regulate Age‐Related Molecule Expression

Promoting the biosynthesis of aging‐related proteins such as collagen and elastin, inhibiting their degradation, can significantly improve the symptoms of lip aging. Peptides, such as palmitoyl tripeptide‐1, palmitoyl pentapeptide‐4, serve as mild and efficient anti‐aging ingredients. Palmitoyl Tripeptide‐1 can promote the synthesis of collagen and glycosaminoglycans, thereby reducing wrinkles. Its activity is comparable to that of retinoic acid, but it is milder and does not cause irritation. Palmitoyl Pentapeptide‐4 can stimulate the production of elastin, fibronectin, glycosaminoglycans, and collagen (especially types I, III, and IV), thereby bolstering the ECM [32].

HA, which is present around and at the interfaces of collagen and elastin fibers, is also a major component of the ECM and has a significant impact on skin aging [33]. Lip anti‐aging products can enhance the moisture content of the lip skin and reduce wrinkles by adding HA. In addition, some plant extracts also have the effect of enhancing skin hydration. For example, perilla extract contains polyphenols, rosmarinic acid, and caffeic acid. These substances can promote the production of hyaluronic acid by upregulating hyaluronic acid synthase 2 (HAS2) and hyaluronic acid synthase 3 (HAS3) and reduce lip roughness [34].

#### 8.1.3 Improve Faded/Dull Lip Color

1. Protect lip blood vessels

Chronic UV exposure triggers dermal ECM degeneration and skin vasculature rarefaction through persistent inflammatory tissue remodeling, leading to age‐related lip color fading [19]. Therefore, in the development of lip products, in addition to the photo‐protection and ECM degradation inhibition mentioned earlier, ingredients with anti‐inflammatory (such as flavonoids, resveratrol, etc.) and vascular function modulation properties (Extracts derived from plants such as *Camellia sinensis, Helichrysum italicum, Artemisia lavandulaefolia*) can also be added simultaneously [35, 36, 37]. These approaches can rebuild the dermal vascular homeostasis micro‐environment through multiple dimensions, thereby delaying age‐related lip color fading.

2 Prevent lip pigmentation

Regarding the dullness of the lips, besides adding sun protection function, lip care products can also incorporate mild whitening ingredients. For example, niacinamide can inhibit melanosome transfer from melanocytes to keratinocytes [38], and tranexamic acid can suppress the production of melanin by reducing prostaglandin synthesis through inhibition of the plasminogen/plasmin system [39, 40], thus improving the dull lip color. As cheilitis may cause post‐inflammatory pigmentation, emphasis should be placed on its prevention. Ingredients with anti‐inflammatory properties can be used to reduce the release of inflammatory factors, prevent post‐inflammatory pigmentation, and restore healthy lip color.

### 8.2 Strengthen the Skin Barrier Function of the Lips

Consumers with sensitive skin and damaged facial skin barriers choose specialized skin care products to repair the skin barrier. The skin barrier of the lips is naturally weak; therefore, consumers should pay attention to strengthening the skin barrier. Skin barrier repair in lip products can be achieved by incorporating cosmetic ingredients that modulate keratinocyte keratinization or by upregulating barrier‐related protein expression. Improving the protective function of the lip‐skin sebum membrane by supplementing or adjusting the content of FFAs, ceramides, and cholesterol, such as by adding lecithin, shea butter, and exogenous ceramides, is also a good choice. Highly occlusive formulations can reduce the TEWL of the lip skin from external sources, improve the moisture content, and resist dry aging. For barrier damage caused by various types of cheilitis, the concomitant use of anti‐inflammatory and soothing agents, such as bisabolol, is essential to control the inflammatory progression and alleviate lip discomfort, facilitating the gradual restoration of skin barrier integrity and preventing chronic cheilitis caused by external factors, thus providing a gentle anti‐aging function.

## 9 Research Gaps and Future Prospects

Although lip aging has been a research hotspot in recent years, there are still certain gaps. First, the molecular mechanisms underlying lip aging remain unclear. Questions such as the role of specific genes in lip aging, the regulatory mechanisms of cell aging‐related signal pathways in the lips, and the differences in cytokines, proteins, and enzyme levels between aged and young lip states require further investigation. Second, there is a lack of data to support the differences in transdermal absorption between lip skin and other body regions. Although the transdermal absorption of the lip skin can be preliminarily speculated based on the theory of transdermal absorption and physiological characteristics of the lip skin, there is a dearth of empirical studies examining the mechanisms, characteristics, and comparative differences with other skin regions. Third, research on the interactions between environmental factors and lip aging is limited. For example, the effects of air pollution, smoking, high altitudes, and extreme temperatures on lip aging are not yet fully understood and require further investigation. Fourth, as cosmetics on the lip vermilion pose a greater risk of oral ingestion, product safety studies, especially on systemic toxicity, need to be conducted.

The future development of lip anti‐aging strategies should focus on more in‐depth, accurate, scientific, and innovative directions. More research should be performed on the aspects of lip aging‐related genes and signaling pathways so that more effective lip anti‐aging ingredients and treatment methods can be developed. In terms of evaluation methods, subjective evaluation, objective instrument measurement, imaging techniques, histochemistry, and molecular biology should be combined to establish a more comprehensive and accurate lip aging evaluation system. In addition, personalized lip anti‐aging treatments for different populations, gender, and age ranges also have great development space and can be formulated according to the characteristics of different consumer needs.

## Conflicts of Interest

The authors declare no conflicts of interest.

## Data Availability

The authors have nothing to report.
